# Integrated Metabolomics and Transcriptomics Reveals Metabolic Pathway Changes in Common Carp Muscle Under Oxidative Stress

**DOI:** 10.3390/antiox14091115

**Published:** 2025-09-14

**Authors:** Yongxiang Liu, Bing Li, Yiran Hou, Linjun Zhou, Qiqin Yang, Chengfeng Zhang, Hongwei Li, Jian Zhu, Rui Jia

**Affiliations:** 1 Wuxi Fisheries College, Nanjing Agricultural University, Wuxi 214081, China; liuyongxiang@stu.njau.edu.cn (Y.L.); lib@ffrc.cn (B.L.); houyr@ffrc.cn (Y.H.); zhoulinjun@ffrc.cn (L.Z.); 2 Key Laboratory of Integrated Rice-Fish Farming Ecology, Ministry of Agriculture and Rural Affairs, Freshwater Fisheries Research Center, Chinese Academy of Fishery Sciences, Wuxi 214081, China; zhangcf@ffrc.cn; 3 Fishery Technology Extension Station of Yunnan, Kunming 650034, China; ynsctgk@163.com; 4 College of Marine Science and Technology and Environment, Dalian Ocean University, Dalian 116023, China; lihw0926@hotmail.com

**Keywords:** hydrogen peroxide, multi-omics, oxidative stress, nutrient quality, metabolic functions, *Cyprinus carpio*

## Abstract

Hydrogen peroxide (H_2_O_2_), a ubiquitous reactive oxygen species in aquatic ecosystems, has been shown to induce toxicological effects in aquatic animals. However, the molecular mechanisms underlying H_2_O_2_-mediated alterations in muscle quality and metabolic homeostasis remain largely unexplored. In this study, we performed integrated metabolomic and transcriptomic analyses to characterize the molecular mechanisms underlying H_2_O_2_-induced oxidative stress in fish muscle tissue. Common carp (*Cyprinus carpio*) were randomized into two groups: a control group (0.0 mM H_2_O_2_) and an H_2_O_2_-treated group (1.0 mM H_2_O_2_) for a 14-day exposure. Following the exposure, comprehensive analyses, including fatty acid composition, amino acid profiles, and multi-omics sequencing, were conducted to elucidate the metabolic responses to oxidative stress. The results showed neither the amino acid nor the fatty acid composition exhibited significant modifications following H_2_O_2_ exposure. Metabolomic profiling identified 83 upregulated and 89 downregulated metabolites, predominantly comprising organic acids and derivatives, lipids and lipid-like molecules. These differential metabolites were associated with histidine and purine-derived alkaloid biosynthesis, glyoxylate and dicarboxylate metabolism pathways. Transcriptomic analysis identified 470 upregulated and 451 downregulated differentially expressed genes (DEGs). GO enrichment analysis revealed that these DEGs were significantly enriched in muscle tissue development and transcriptional regulatory activity. KEGG analysis revealed significant enrichment in oxidative phosphorylation, adipocytokine signaling, and PPAR signaling pathways. The elevated oxidative phosphorylation activity and upregulated adipocytokine/PPAR signaling pathways collectively indicate H_2_O_2_-induced metabolic dysregulation in carp muscle. Through the integration of metabolomics and transcriptomics, this study offers novel insights into the toxicity of H_2_O_2_ in aquatic environments, elucidates adaptive mechanisms of farmed fish to oxidative stress, and provides a theoretical basis for developing antioxidant strategies.

## 1. Introduction

Hydrogen peroxide (H_2_O_2_), a ubiquitous reactive oxygen species (ROS) in aquatic ecosystems, plays dual roles in environmental and aquaculture applications [1]. Naturally occurring in various water bodies (ponds, rivers, lakes, and oceans), H_2_O_2_ primarily originates from photochemical reactions involving dissolved organic matter (DOM) [1,2,3]. As a key redox intermediate, it significantly influences aquatic biogeochemical cycles and water quality parameters [4]. This compound has demonstrated practical utility in water management, where controlled doses effectively suppress cyanobacterial blooms [5] and improve water quality [6]. In aquaculture practices, H_2_O_2_ has been widely implemented as a therapeutic agent against fungal, bacterial, and parasitic pathogens that threaten fish health [7]. Its application in recirculating aquaculture systems (RAS) requires careful consideration—while low concentrations in biofilters can impair ammonium removal and nitrification processes [8], strategic dosing in protein skimmers may simultaneously protect biofilter integrity and maintain beneficial microbial communities critical for fish cultivation [9].

H_2_O_2_ is a well-documented source of ROS, routinely employed to establish oxidative stress model in both in vitro and in vivo experimental systems [10,11,12,13]. It mediates oxidative damage through multiple mechanisms, such as overwhelming cellular antioxidant defenses and disrupting essential signaling pathways [14]. Research has demonstrated that H_2_O_2_ exposure suppressed thioredoxin-2 (TXN2) expression in adipocytes while concurrently activating the NF-κB signaling cascade [12]. In muscle tissue, H_2_O_2_-induced oxidative stress triggers the CaMKK/LKB1/AMPK pathway, resulting in redox system imbalance, impaired energy metabolism, glycolytic flux enhancement, and ultimately reduced growth performance [15]. H_2_O_2_ exposure differentially alters protein expression profiles in two rat salivary acinar cell lines, particularly affecting proteins associated with mitochondrial function, apoptotic regulation, and chromatin remodeling [16].

In aquatic animals, H_2_O_2_ toxicity has been widely documented. Prolonged exposure to elevated H_2_O_2_ concentrations can trigger non-specific immune responses, induce oxidative stress, and cause physiological damage in farmed fish [17]. Prior research has revealed that H_2_O_2_ treatment caused tissue-specific impairments in common carp, including ion transport dysfunction in gills [18], as well as metabolic disturbances, inflammatory responses, and cytotoxicity in liver tissues [19]. Existing studies have shown that H_2_O_2_ can cause tissue-specific damage in various aquatic animals. In the liver of *Monopterus albus*, it induced oxidative stress, inflammatory responses, and apoptosis [20,21]. Additionally, in *Thamnaconus septentrionalis*, it elicited multiple pathological effects, including damage to the intestinal mucosa, immune-inflammatory responses, and energy metabolism disorders [22].

As the primary edible part of fish, muscle tissue is rich in high-quality protein, polyunsaturated fatty acids (such as EPA and DHA), and essential trace elements [23]. These nutrients play crucial roles in supporting human health, nervous system development, and immune function regulation [24,25,26]. The quality of fish muscle—which can be affected by lipid oxidation and protein oxidation—directly influences the commercial value of aquatic products and poses potential risks to consumer health [27,28]. Therefore, investigating oxidative stress in muscle tissue has direct economic and food safety implications. Moreover, muscle tissue exhibits a unique energy metabolism profile, including glycogen storage and glycogenolysis, along with a high content of lipids rich in oxidation-sensitive unsaturated fatty acids [29,30]. These compositional and metabolic features make it highly vulnerable to oxidative damage and potentially a more representative indicator of the organism’s overall health status. Nevertheless, the oxidative damage mechanisms in fish muscle tissue remain poorly understood. Therefore, this study employed metabolomics and transcriptomics approaches to assess H_2_O_2_-induced oxidative stress in the muscle tissues of common carp and elucidate the underlying molecular mechanisms. Integrating transcriptomic and metabolomic analyses aims to establish correlations between differentially expressed genes and differentially accumulated metabolites, thereby enabling a systematic dissection of the mechanisms underlying the oxidative stress response. Our findings provide novel insights into the responsive mechanisms of cultured fish to H_2_O_2_ exposure, contributing to more accurate risk assessment of H_2_O_2_ in aquatic environments.

## 2. Materials and Methods

### 2.1. Fish, Experimental Design and Sampling

Common carp (*Cyprinus carpiovar Jian*, mean weight = 159.65 ± 15.99 g) were sourced from the farm of Freshwater Fisheries Research Center (Wuxi, China). Healthy specimens were acclimatized for 7 days in recirculating aquaculture systems under controlled conditions: water temperature 24–26 °C, pH 7.3–7.6, and DO > 5.0 mg/L. During acclimation, the fish were fed twice daily (08:30 and 15:30) with a commercial diet (Tongwei, Wuxi, China; crude protein ≥ 29.00%, crude fat ≥ 4.5%, crude fiber ≤ 12.0%, ash ≤ 15.0%) at 2% of their weight.

**Figure 1:**
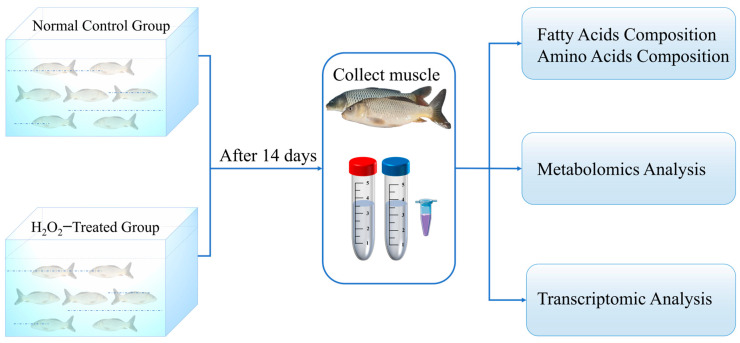
Workflow diagram of the experimental design.

Following acclimation, the carp were randomized into two experimental groups: normal control (NC group, 0 mM H_2_O_2_) and H_2_O_2_ treatment (HT group, 1.0 mM H_2_O_2_). Each group consisted of 21 fish divided into 3 replicates (*n* = 7 per replicate). The experiment lasted for 14 days, during which fish were exposed to H_2_O_2_ for 1 h daily. Throughout the experimental period, fish were fed appropriate amounts of feed to avoid starvation-induced stress. The recirculating aquaculture system was supplied with fully aerated drinking water, and key water quality parameters were maintained as follows: DO > 5.0 mg/L, pH 7.3–7.6, NO_2_^−^ < 0.02 mg/L, and NH_3_ < 0.05 mg/L. At the end of the exposure period, fifteen fish were randomly sampled from each group (5 fish per tank, 3 tanks per group) and immediately anesthetized with MS-222 (100 mg/L; Sigma, St. Louis, MO, USA). Dorsal muscle tissues from every three fish were pooled to form one composite sample, yielding five biologically independent replicates per group. These samples were used for integrated multi-omics analyses, including metabolomics, transcriptomics, and composition profiling of amino acids and fatty acids. Following immediate freezing in liquid nitrogen, the samples were transported on dry ice to Gene Denovo (Guangzhou, China) for subsequent sequencing analysis. Throughout the study, personnel involved in sample processing and data acquisition were blinded to group assignments. All procedures involving animals were carried out in compliance with animal welfare guidelines and were approved by the Freshwater Fisheries Research Center. No mortality was observed in either the NC or HT groups throughout the experimental period. Figure 1 displays a schematic of the experimental workflow.

### 2.2. Measurement of Amino Acids and Fatty Acids in Muscle

According to the standard method (Chinese National Standard (GB5009.124-2016) [31]), muscle tissue samples (100 mg wet weight) were digested in 10 mL of 6 M hydrochloric acid at 110 °C for 22 h using sealed hydrolysis tubes. After hydrolysis, the solution was filtered into a 50 mL volumetric flask. A 1.0 mL aliquot of the filtrate was transferred to a 15 mL test tube and concentrated under reduced pressure at 40 °C. The dried residue was then reconstituted in 1.0 mL of sodium citrate buffer (pH 2.2) and filtered through a 0.22 μm membrane. Finally, quantitative amino acid analysis was performed using a Hitachi amino acid analyzer (Tokyo, Japan).

A 200 mg muscle tissue was mixed with 10 mL of 8.3 M HCL and hydrolyzed at 70–80 °C for 40 min. According to the standard method (Chinese National Standard (GB5009.168-2016) [32]), 30 mL of a mixed solution of diethyl ether and petroleum ether (1:1, *v*/*v*) was added to the hydrolysate for total lipid extraction. Lipid methylation was performed with 14% BF_3_-methanol at 45 °C (20 min) to generate fatty acid methyl esters (FAMEs). Subsequently, gas chromatography (GC) analysis was conducted using an Agilent 7890A system (Santa Clara, CA, USA) fitted with an HP-88 column under conditions (injector: 250 °C; detector: 260 °C). FAME standards (Sigma, St. Louis, MO, USA) served as references for fatty acid composition.

### 2.3. Non-Targeted Metabolome Analysis

Tissue samples (100 mg) from each group were homogenized in 1 mL of pre-chilled ternary solvent (methanol/acetonitrile/water, 2:2:1) and subsequently centrifuged (13,000× *g*, 15 min, 4 °C). The supernatant was analyzed using a UHPLC system (1290 Infinity LC, Agilent Technologies, Santa Clara, CA, USA), and mass spectra (both MS1 and MS2) were acquired using an TripleTOF 6600 mass spectrometer (Sciex, Shanghai, China).

The ESI source conditions were set as follows: Ion Source Gas 1 (Gas1): 60, Ion Source Gas 2 (Gas2): 60, Curtain Gas (CUR): 30, Source Temperature: 600 °C, and Ion Spray Voltage Floating (ISVF): ±5500 V (pos and neg modes). The TOF MS scan range was *m*/*z* 60–1000 Da, and the product ion scan range was *m*/*z* 25–1000 Da. The accumulation time was 0.20 s per spectrum for TOF MS scans and 0.05 s per spectrum for product ion scans. Quality control (QC) samples were prepared by pooling equal volumes from each test sample and were used to monitor system stability and evaluate the reliability of the experimental data. Raw data were transformed to .mzML format, followed by metabolite annotation using XCMS software (version 3.7.1). Metabolite profiling employed complementary ion modes (positive and negative) to achieve enhanced coverage. Orthogonal partial least squares-discriminant analysis (OPLS-DA) was applied to distinguish metabolomic profiles between the two groups. Differentially expressed metabolites (DEMs) between NC and HT groups were identified based on variable importance in projection (VIP) scores from the OPLS-DA model, with thresholds set at VIP ≥ 1 and *p*-value < 0.05. KEGG (Release 101) pathway enrichment analysis was performed by database mapping of DEMs.

### 2.4. Transcriptome Analysis

Following mRNA enrichment (mRNA Capture Beads) of NC and HT samples, fragmented transcripts were reverse-transcribed. The resulting ds cDNA underwent purification and PCR-based library preparation, with final sequencing performed via Illumina NovaSeq X Plus (Gene Denovo, Guangzhou, China).

Raw reads were quality-filtered using FASTP (version 0.18.0) to generate clean reads. Short reads alignment tool Bowtie2 (version 2.2.8) was used for mapping reads to ribosome RNA (rRNA) database. The rRNA-mapped reads will then be removed. The remaining clean reads were aligned to the *Cyprinus carpio* reference genome (NCBI accession: GCF_000951615.1) with HISAT2 (version 2.1.0) under default parameters. DESeq2 (version 1.20.0) was employed to identify differentially expressed genes (DEGs) between the NC and HT groups, applying significance thresholds of FDR < 0.05 and |log_2_ FC| ≥ 1. The resulting DEGs were subsequently annotated against GO (3.14.0) and KEGG (Release 101) pathway databases for functional characterization. GSEA (version 2.2.4) was further employed to identify significantly altered pathways, using the following criteria: |Normalized Enrichment Score (ES)| > 1, nominal *p*-value < 0.05, and FDR < 0.25.

To validate the transcriptome sequencing results, qPCR analysis was performed using gene-specific primers (listed in Table S1). Key genes were selected from significantly enriched pathways for validation, based on their crucial roles in metabolic functions. Total RNA was extracted from muscle tissues with RNAiso Plus reagent (Takara, Beijing, China) and reverse-transcribed into cDNA using the PrimeScript™ RT reagent kit (Takara). qPCR amplification was conducted with TB Green Premix Ex Taq II (Takara, RR820A) using cDNA as the template. Three technical replicates were performed for each sample. The quantification cycle (Cq) values were analyzed, and relative gene expression levels were calculated using the 2^−ΔΔCq^ method, with β-actin and 18S rRNA as reference genes.

### 2.5. Statistical Analysis

Using SPSS Statistics software (version 27.0), all quantitative data were analyzed and reported as mean ± SEM. Intergroup variations in amino acid and fatty acid profiles were evaluated using independent *t*-tests, adopting *p* < 0.05 as the significance threshold for NC versus HT group comparisons. Normality of all variables was assessed using the Shapiro–Wilk test, and homogeneity of variances was verified by Levene’s test. If both normality and homoscedasticity were satisfied, an independent samples *t*-test was applied; otherwise, Welch’s corrected *t*-test was used. The data were analyzed blindly with group assignments concealed from the statistician until all analyses were finalized. Pearson correlation analysis was performed to evaluate the consistency between qPCR and RNA-seq results, with statistical significance defined as *p* < 0.05.

## 3. Results

### 3.1. Amino Acid and Fatty Acid Composition in Muscle

In the muscle tissue, 17 amino acids were identified, comprising 7 essential amino acids (EAAs), 2 conditionally essential amino acids (CEAAs), and 8 non-essential amino acids (NEAAs). Although no statistically significant differences were observed in amino acid content between the NC and HT groups, percentage difference analysis indicated a declining trend in all categories of amino acids in common carp muscle following H_2_O_2_ exposure, with essential amino acids decreasing by 5.35% and non-essential amino acids by 6.50% (Table 1).

Twelve fatty acids were detected in carp muscle, consisting of 2 saturated fatty acids (SFAs) and 10 unsaturated fatty acids (UFAs). H_2_O_2_ treatment showed decreased levels of most fatty acids, with the exception of C20:2, C22:1n9, and C22:6n3, which exhibited increased concentrations. However, fatty acid levels did not differ significantly between the groups (*p* > 0.05). H_2_O_2_ treatment reduced the contents of C16:1 and C18:3n3 in muscle by 17.27% and 10.50%, respectively, and decreased the total unsaturated fatty acid content by 7.91% (Table 2).

### 3.2. Metabolomics Analysis in Muscle

#### 3.2.1. Metabolite Identification

**Figure 2:**
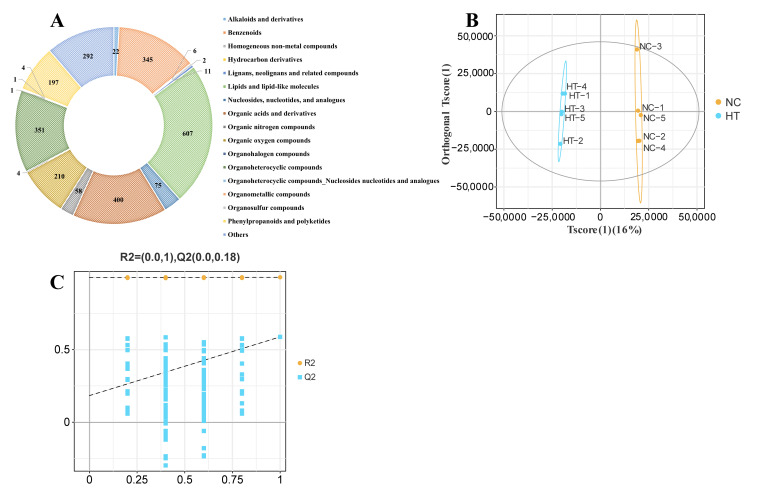
Metabolome analysis of muscle after H_2_O_2_ exposure: (**A**) Numbers and classification of the annotated metabolites. (**B**) OPLS-DA analysis of samples in NC and HT groups. (**C**) Permutation Test Plot of the OPLS-DA.

Metabolomic profiling identified 2586 metabolites in muscle tissues (Figure 2A). OPLS-DA showed distinct clustering patterns of metabolites between the two groups (Figure 2B), and cross-validation with permutation tests confirmed the reliability of the OPLS-DA model (Figure 2C).

#### 3.2.2. Metabolite Differential Analysis

**Figure 3:**
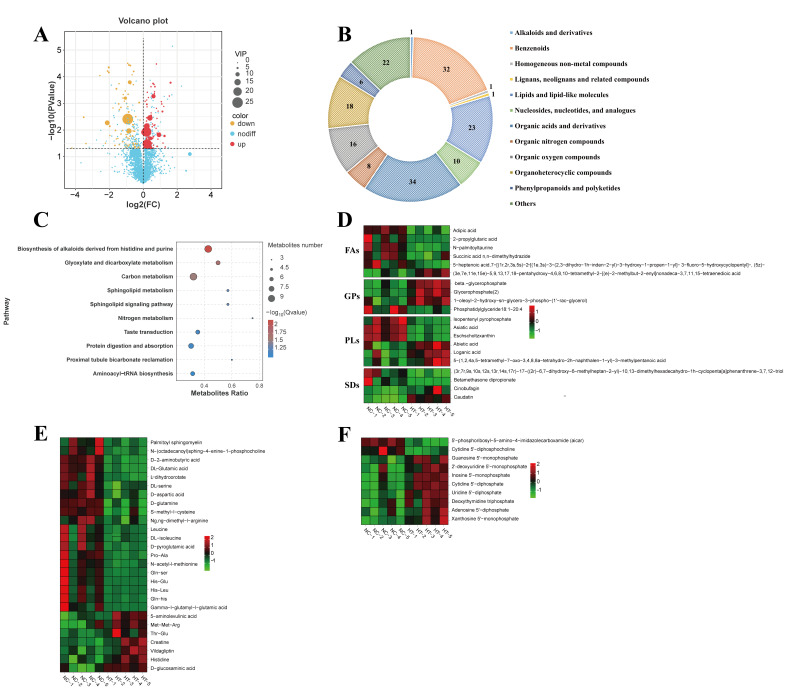
DEMs in the muscles of *C. carpio* after H_2_O_2_ exposure: (**A**) Volcano plot of the DEMs between NC and HT groups. (**B**) Numbers and classification of the DEMs. (**C**) Main KEGG pathways. (**D**) DEMs related to lipids and lipid-like molecules (FA, fatty acyls; GP, glycerophospholipids; PL, prenol lipids; SD, steroids and steroid derivatives). (**E**) DEMs related to amino acids, peptides, and analogues. (**F**) DEMs related to nucleosides, nucleotides, and analogues.

H_2_O_2_ treatment caused significant abundance changes in 172 metabolites (83 upregulated, 89 downregulated) (Figure 3A, VIP ≥ 1 and *p*-value < 0.05). The major DEMs were categorized into the following classes: organic acids and derivatives (34), benzenoids (32), lipids and lipid-like molecules (23), organoheterocyclic compounds (18), and organic oxygen compounds (16) (Figure 3B).

KEGG enrichment analysis revealed that the DEMs showed a predominant statistical connection with: biosynthesis of alkaloids derived from histidine and purine (*q* = 0.0065), glyoxylate and dicarboxylate metabolism (*q* = 0.0186), and carbon metabolism (*q* = 0.0278) (Figure 3C).

Significant changes were identified in lipid-related DEMs, including 6 pregnenolone lipids (PRs), 6 fatty acyls (FAs), 4 glycerophospholipids (GPs), and 4 steroids and steroid derivatives between NC and HT groups (Figure 3D). Among FAs, HT treatment significantly decreased five metabolites (adipic acid, N-palmitoyltaurine, etc.) while increasing one metabolite (*p* < 0.05). For PRs, H_2_O_2_ exposure elevated three metabolites (asiatic acid, eschscholtzxinanthin, isopentenyl pyrophosphate) and reduced three others (loganic acid, abietic acid, etc.) (*p* < 0.05). Notably, phosphatidylglycerol (18:1–20:4) was significantly suppressed by H_2_O_2_ treatment among GPs (*p* < 0.05).

A significant alteration in the concentrations of 25 amino acids and associated derivatives was observed upon H_2_O_2_ exposure (Figure 3E). Compared to the NC group, 18 compounds—including DL-glutamic acid, DL-serine, D-aspartic acid, D-glutamine, and γ-L-glutamyl-L-glutamic acid—were significantly reduced, whereas 7 others (e.g., histidine, creatine, and Met-Met-Arg) exhibited increased levels (*p* < 0.05).

H_2_O_2_ exposure significantly altered relative abundance of nucleotides and their analogs in muscle tissue, elevating eight compounds, including inosine 5′-monophosphate (IMP), adenosine 5′-diphosphate (ADP), and uridine 5′-diphosphate (UDP), while reducing cytidine 5′-diphosphocholine (CDP-choline) levels (*p* < 0.05; Figure 3F).

### 3.3. Transcriptomic Analysis in Muscle

#### 3.3.1. Differential Analysis

After raw data filtering, >98.9% valid data were obtained. The transcriptome sequencing yielded 5,507,688,984–7,249,214,727 bp of clean reads, with Q_20_ and Q_30_ base percentages exceeding 97.2% and 94.2%, respectively, in both groups. The GC content ranged between 49.05 and 50.04%. The reliability of the sequencing data obtained from muscle tissues in both NC and HT groups was demonstrated by these results. (Table 3).

**Figure 4:**
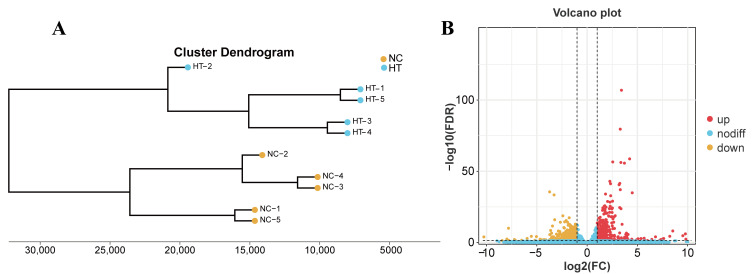
DEGs in the muscle of *C. carpio* (NC vs. HT): (**A**) Cluster dendrogram of NC and HT groups. (**B**) Volcano plot of DEGs in the RNA-seq.

The cluster dendrogram revealed clear separation between NC and HT groups, indicating significant alterations in gene expression patterns after H_2_O_2_ treatment in carp muscle tissue (Figure 4A). Differential expression analysis identified 921 DEGs after H_2_O_2_ treatment, comprising 470 upregulated and 451 downregulated genes (Figure 4B, FDR < 0.05 and |log_2_ FC| ≥ 1).

#### 3.3.2. Enrichment Analysis of GO and KEGG

**Figure 5:**
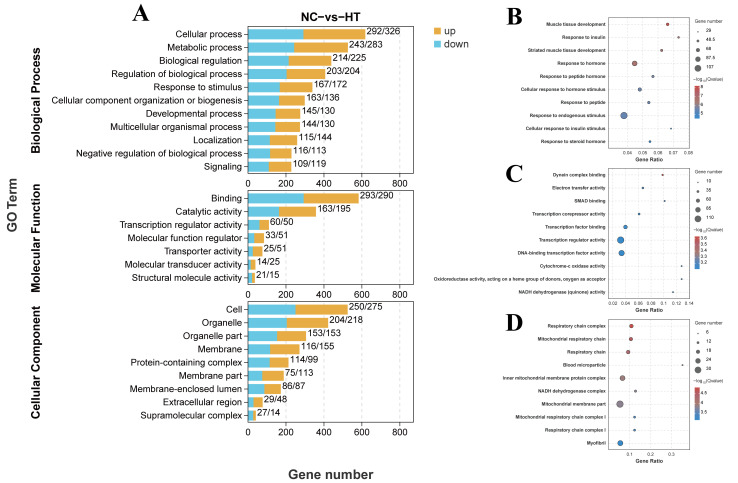
GO analysis for the DEGs in the muscle of *C. carpio* (NC vs. HT): (**A**) GO terms of DEGs in level 1 and level 2. (**B**) The top 10 GO terms in the biological process category. (**C**) The top 10 GO terms in the molecular function category. (**D**) The top 10 GO terms in the cellular component category.

To elucidate the biological effects of H_2_O_2_ exposure on carp muscle, we performed GO analysis for DEGs, which were distributed across biological processes, molecular functions, and cellular components (Figure 5A). Notably, DEGs showed strong association with muscle tissue development (*p* adjust < 0.001) among biological processes (Figure 5B). For molecular functions, DEGs were predominantly associated with transcription regulator activity (*p* adjust = 0.008) (Figure 5C). Cellular component analysis revealed significant enrichment in blood microparticles (*p* adjust = 0.0001) and myofibrils (*p* adjust = 0.007) (Figure 5D).

**Figure 6:**
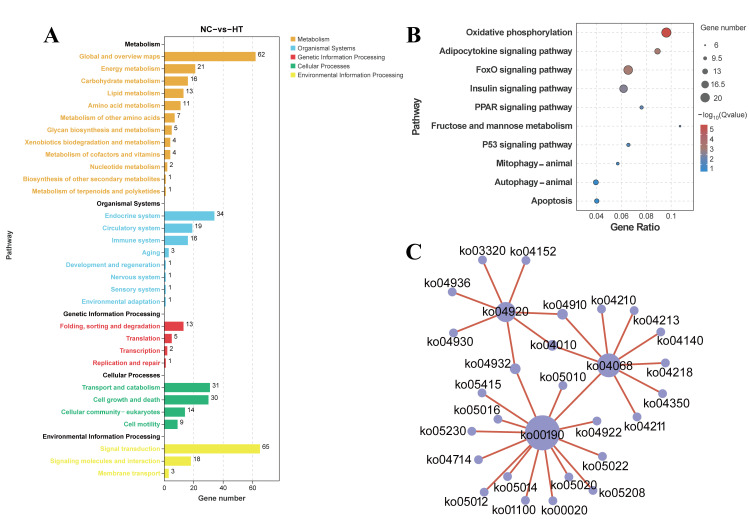
KEGG enrichment analysis of *C. carpio* muscle (NC vs. HT): (**A**) DEGs enrichment in the KEGG Class A and B. (**B**) The top 10 enriched KEGG pathways. (**C**) Network plot of the interactions after H_2_O_2_ exposure.

The KEGG pathway enrichment analysis revealed significant enrichment across five major categories: organismal systems, metabolism, and environmental information processing being the most prominent (Figure 6A). The top enriched pathways included: oxidative phosphorylation (*q* < 0.0001), adipocytokine signaling pathway (*q* = 0.0020), FoxO signaling pathway (*q* = 0.0020), insulin signaling pathway (*q* = 0.0080), PPAR signaling pathway (*q* = 0.0387), and fructose and mannose metabolism (*q* = 0.0387) (Figure 6B,C).

#### 3.3.3. Alterations of Pathways of FoxO and Insulin

**Figure 7:**
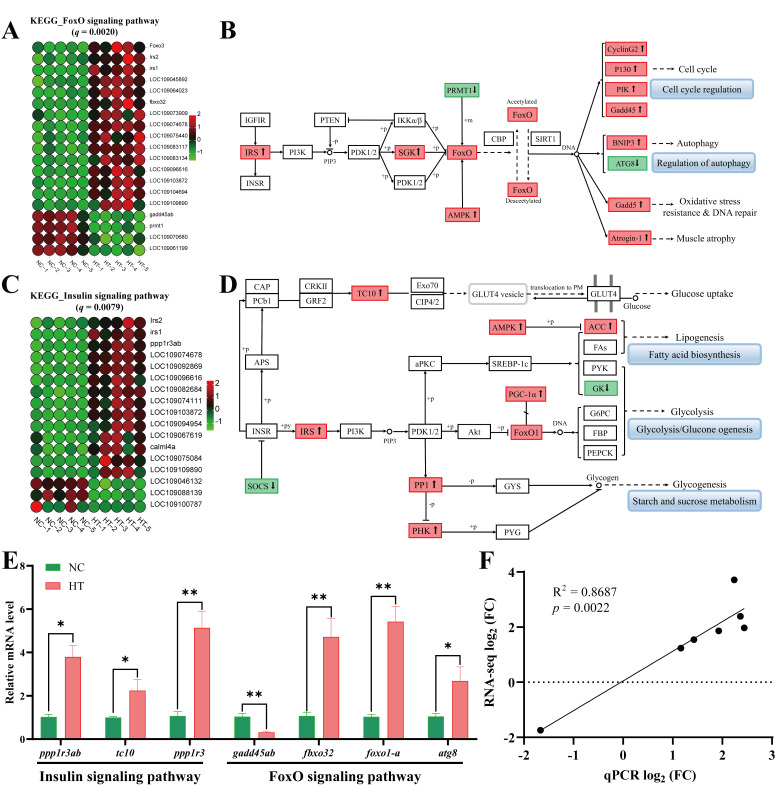
Changes in the FoxO and Insulin signaling pathways in *C. carpio* muscle after H_2_O_2_ exposure: (**A**) Heatmap of DEGs in the FoxO signaling pathway. (**B**) FoxO signaling pathway diagram in the KEGG (⬆: up, ⬇: down). (**C**) Heatmap of DEGs in the insulin signaling pathway. (**D**) Insulin signaling pathway diagram in the KEGG (⬆: up, ⬇: down). (**E**) qPCR analysis of key genes in FoxO and insulin signaling pathways, with values expressed as the mean ± SEM (*n* = 5), * *p* < 0.05 and ** *p* < 0.01. (**F**) The correlation between the results from qPCR and RNA-seq.

In the FoxO signaling pathway, H_2_O_2_ exposure significantly upregulated 15 genes and downregulated 4 genes (Figure 7A). Pathway analysis revealed that H_2_O_2_ activates FoxO through upregulation of IRS, AMPK, and SGK, subsequently increasing expression of P130, BNIP3, Gadd45, and Atrogin-1, which collectively contribute to muscle atrophy, autophagy, oxidative stress, and DNA damage (Figure 7B). Within the insulin signaling pathway, H_2_O_2_ treatment upregulated 14 genes and downregulated 3 genes (Figure 7C). Mechanistically, H_2_O_2_ elevated expression of IRS, PHK, PP1, AMPK, and FoxO1 in carp muscle tissue, which modulated ACC and GK expression to directly or indirectly activate lipid biosynthesis and glucose metabolism pathways (Figure 7D). Furthermore, qPCR validation of key genes associated with the FoxO and insulin signaling pathways (Figure 7E) demonstrated significant consistency between RNA-seq and qPCR data (R^2^ = 0.8687, *p* = 0.0022; Figure 7F).

#### 3.3.4. Alterations of Metabolism-Related Pathways

**Figure 8:**
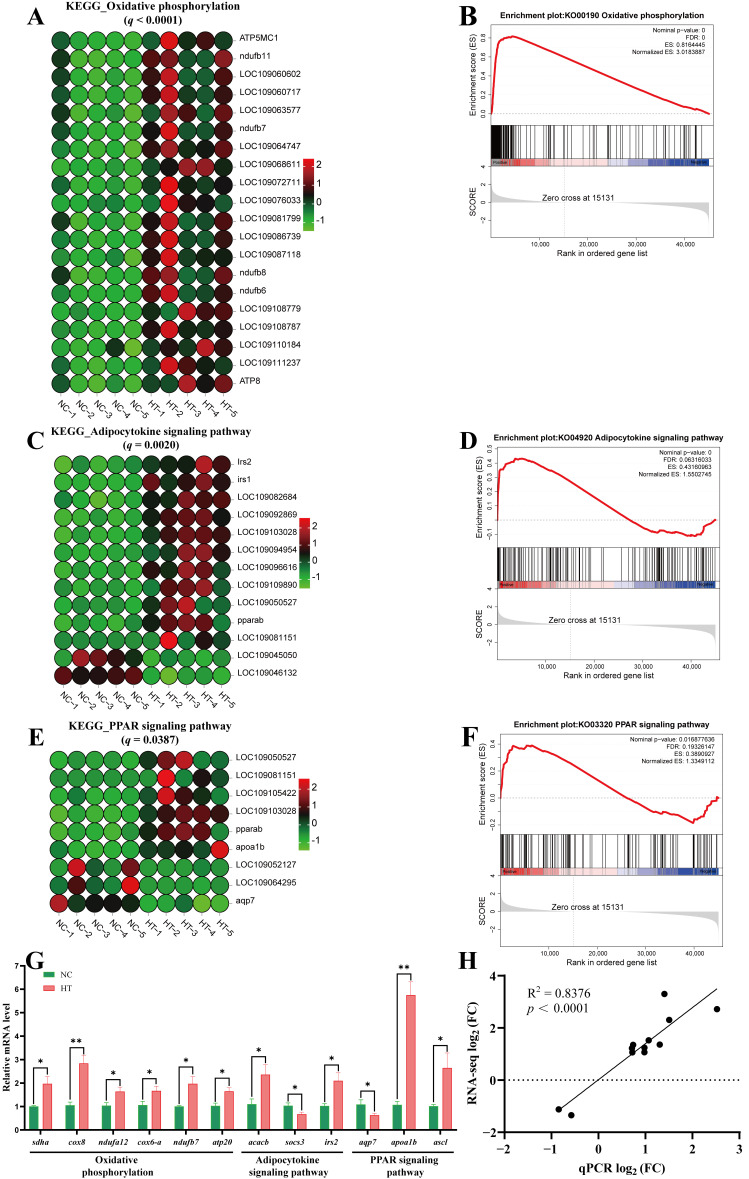
Changes in key metabolic pathways in muscle tissue after H_2_O_2_ exposure: (**A**) Heatmap of DEGs in the oxidative phosphorylation pathway. (**B**) GSEA for the oxidative phosphorylation pathway. (**C**) Heatmap of DEGs in the adipocytokine signaling pathway. (**D**) GSEA for the adipocytokine signaling pathway. (**E**) Heatmap of DEGs in the PPAR signaling pathway. (**F**) GSEA for the PPAR signaling pathway. Statistical significance for each gene set was defined by the following threshold criteria: the |Normalized ES| > 1, nominal *p*-value < 0.05 and FDR < 0.25. (**G**) Expression of key genes related to the metabolic pathway (qPCR validation); values are expressed as mean ± SEM (*n* = 5), * *p* < 0.05 and ** *p* < 0.01. (**H**) The correlation between the results from qPCR and RNA-seq.

Transcriptomic analysis revealed distinct H_2_O_2_-induced alterations in key metabolic pathways. In oxidative phosphorylation pathway, H_2_O_2_ exposure significantly upregulated 20 genes (Figure 8A). In the adipocytokine signaling pathway, H_2_O_2_ treatment upregulated 11 genes and downregulated 2 genes (Figure 8C). Compared to the NC group, the HT group showed upregulation of 6 genes and downregulation of 3 genes in the PPAR signaling pathway (Figure 8E). Further, significant positive enrichment of these three pathways was demonstrated by GSEA under H_2_O_2_-induced oxidative stress. (Figure 8B,D,F). Additionally, we validated the expression of key metabolic pathway genes showing significant alterations (including *sdha*, *cox8*, *atp20*, *socs3*, and *apoa1*) by qPCR (Figure 8G). The results exhibited strong positive correlation with RNA-seq data (R^2^ = 0.8376, *p* < 0.001; Figure 8H).

## 4. Discussion

H_2_O_2_, a potent oxidizing agent, can induce oxidative stress and physiological damage in farmed fish upon chronic exposure. Previous studies have demonstrated that H_2_O_2_ impaired hepatic function and triggered apoptosis and inflammatory responses in fish. Under the present experimental conditions, we assessed the influences of H_2_O_2_ on the amino acid and fatty acid profiles of common carp and elucidated the underlying mechanisms of oxidative stress through integrated metabolomic and transcriptomic analyses.

### 4.1. The Effect of Oxidative Stress on Muscle Nutrient Quality

Fish muscle is characterized by low fat content and high nutritional value, being rich in high-quality proteins, essential amino acids, and n-3 PUFAs, particularly DHA and EPA [23]. These components render fish a premium source of essential nutrients for human health.

The composition and homeostasis of amino acids in muscle tissue are critical determinants of nutrient quality, which are modulated by both dietary sources and environmental conditions [33]. Dietary EAA supplementation directly influences peripheral tissues (skeletal muscle, adipose tissue, and liver) to coordinate metabolic and energy homeostasis, prevent oxidative damage, and enhance immune function [34,35,36]. The total hydrolyzed amino acid content of *Crassostrea oysters* decreased significantly over time after exposure to hypersaline conditions [37]. Under high-density culture stress, the muscle tissues of common carp exhibit a significant decrease in glycine (Gly) [38]. Our findings indicated non-significant alterations in the muscle amino acid profile of common carp following H_2_O_2_ exposure, although moderate decreases in essential and conditionally essential amino acids were observed. These results are consistent with previous reports showing that density stress also induced non-significant changes in amino acid content in the snail *Bellamya purificata* [39].

Fatty acids serve as fundamental nutritional determinants of muscle quality in aquaculture species, where their compositional profile and abundance directly influence both nutritional value and organoleptic characteristics of fish flesh [40,41]. Previous studies have demonstrated that physiological stress significantly modulates lipid metabolic pathways in fish [42]. As fundamental constituents of cell membranes, unsaturated fatty acids (UFAs) are critically involved in regulating energy metabolism. For example, kuruma shrimp (*Marsupenaeus japonicus*) upregulate UFA biosynthesis to maintain membrane fluidity under cold stress conditions [43]. Similarly, high-density culture stress has been shown to induce significant increases in saturated fatty acids, monounsaturated fatty acids, n-3 PUFAs, and n-6 PUFAs in common carp muscle [38]. However, our study found non-significant differences in the fatty acid profile of carp muscle after H_2_O_2_ exposure, suggesting H_2_O_2_-induced oxidative stress may not significantly alter the fatty acid composition in carp muscle tissue. These results further imply that such exposure likely has minimal impact on the muscle quality of carp.

### 4.2. The Effects of Oxidative Stress on Muscle Metabolite Composition

Given the strong correlation between muscle quality and metabolite profiles, metabolomics has become a powerful instrument for elucidating the molecular mechanisms underlying changes in muscle quality in aquatic organisms exposed to various stress conditions [23,44,45]. Substantial evidence demonstrates that environmental stressors significantly disrupt key metabolic pathways, including glucose, lipid metabolism, and amino acid metabolism in oriental river prawn (*Macrobrachium nipponense*) [46], rainbow trout (*Oncorhynchus mykiss*) [47], and. gilthead sea bream (*Sparus aurata*) [48].

Lipids are essential components for maintaining homeostasis, physiological functions, and cellular integrity in animals [49,50]. Beyond their structural roles, emerging evidence highlights their regulatory functions in signaling pathways, immune modulation, and stress adaptation [51,52,53,54]. Our study identified significant perturbations in lipid metabolism in common carp muscle under H_2_O_2_ exposure, characterized by the upregulation of adipic acid, 2-propylglutaric acid, N-palmitoyltaurine, phosphatidylglycerol (18:1–20:4), glycerophosphate, and β-glycerophosphate, alongside the downregulation of isopentenyl pyrophosphate, asiatic acid, and betamethasone dipropionate. These alterations likely reflect a coordinated adaptive response to oxidative stress. The upregulation of adipic acid—an intermediate in fatty acid β-oxidation—suggests altered mitochondrial energy metabolism [55]. The observed changes in glycerophospholipids (e.g., phosphatidylglycerol) are particularly noteworthy, as these molecules are critical for maintaining membrane fluidity and signaling capacity [38,56,57]. Thermal and cold stress studies in turbot (*Scophthalmus maximus*) and yellow drum (*Nibea albiflora*) demonstrate that glycerophospholipid metabolism is highly sensitive to environmental stressors [58,59]. In rainbow trout, heat-induced disruption of glycerophospholipid homeostasis correlates with hepatocellular damage [60], while cold stress reduces phosphatidylethanolamine and phosphatidylcholine in red swamp crayfish [61]. Our findings aligned with these reports, suggesting that common carp may remodel membrane lipid composition to preserve cellular integrity under oxidative stress. Specifically, the accumulation of polyunsaturated phosphatidylglycerol (18:1–20:4) may represent an attempt to maintain membrane fluidity.

In aquaculture, environmental stressors frequently disrupt amino acid homeostasis and compromise antioxidant defenses in aquatic species, ultimately exacerbating oxidative damage [62,63]. For instance, under water flow stress, the muscle tissue of largemouth bass showed significant alterations in amino acid metabolites, such as L-glutamate, L-isoleucine, L-arginine, L-tyrosine, and L-phenylalanine, affecting related metabolic pathways [64]. During cold stress, Pacific white shrimp (*Litopenaeus vannamei*) exhibited increased levels of key amino acids like proline, alanine, glutamate, and taurine in their hepatopancreas to bolster energy metabolism [65]. Furthermore, amino acid metabolism has been shown to participate in metabolic regulation and osmoregulation maintenance in sea cucumbers under hypoxic stress [66]. In this study, exposure to H_2_O_2_ led to significant reductions in glutamate-related metabolites, specifically DL-glutamic acid, D-glutamine, and γ-L-glutamyl-L-glutamic acid. Concurrently, there were marked increases in histidine, its dipeptides (His-Glu, His-Leu), and creatine in muscle tissue. Glutamate functions as both a pivotal precursor for amino acid biosynthesis and a critical anaplerotic substrate for the TCA cycle, and its depletion may reflect impaired mitochondrial energy metabolism in muscle tissue [64]. Furthermore, the reduction in γ-L-glutamyl-L-glutamic acid, a glutathione precursor, may compromise the organism’s antioxidant capacity [67,68]. The antioxidant properties of histidine have been well-documented, and its elevated levels may mitigate oxidative damage through free radical scavenging [69,70,71]. This observation potentially correlates with the histidine-derived alkaloid biosynthesis pathway identified in KEGG enrichment analysis, further supporting histidine’s crucial role in oxidative defense. In Cr-PCr-CK system, creatine mediates the interconversion between phosphocreatine and ATP under the catalysis of creatine kinase, thereby buffering cellular energy supply [72,73]. As a central molecule in muscular energy buffering systems, increased creatine levels may help maintain ATP homeostasis to counteract energy metabolism dysregulation induced by oxidative stress [74]. Collectively, these alterations in amino acid metabolites suggest that muscle tissue undergoes metabolic reprogramming and adaptive adjustments to maintain functional stability in response to oxidative stress.

Dysregulation of nucleotide metabolites might contribute to muscle functional abnormalities and defective repair processes [75]. Glyphosate exposure has been shown to dysregulate nucleotide metabolic pathways in grass carp (*Ctenopharyngodon idellus*) muscle, inducing oxidative stress and systemic metabolic disturbances [75]. Nitrite exposure has been shown to impair nucleotide metabolism in Pacific white shrimp, resulting in significant decreases in nucleic acid derivatives, including guanosine, inosine, and thymidine [76]. Furthermore, H_2_O_2_ induces oxidative stress in cardiomyocytes and alters nucleotide metabolism [77]. Our results expand on these findings by showing that oxidative stress leads to the accumulation of ATP catabolites, particularly IMP and GMP. The elevated levels of these catabolites provide direct evidence of accelerated ATP turnover under oxidative conditions, which reflects an increased energy demand during cellular stress responses. These nucleotide derivatives, along with other metabolic intermediates, may be released as byproducts of cellular stress responses or as components of adaptive mechanisms, potentially exerting significant modulatory effects on cellular signaling cascades [78].

### 4.3. The Effects of Oxidative Stress on Muscle Metabolism-Related Pathways

Environmental stressors have been unequivocally shown to induce oxidative stress that disrupts metabolic homeostasis in aquatic organisms [79]. This stress response coordinately activates multiple signaling pathways that mediate metabolic reprogramming and cellular adaptation. Under thermal stress, significant alterations are observed in the expression profiles of the PPAR and adipocytokine signaling pathways in the muscle tissue of *Schizothorax wangchiachii* [80]. Similarly, acute hypoxic stress activates the insulin and PPAR signaling pathways in the liver tissue of largemouth bass, thereby regulating its metabolic adaptation processes [81]. Furthermore, the FoxO signaling pathway serves as a key regulator in the anti-stress defense mechanisms of mud crabs against nitrite stress [82]. In line with previous studies, transcriptomic KEGG enrichment analysis in this study indicated that H_2_O_2_ exposure significantly altered multiple metabolic pathways in carp muscle, including FoxO signaling, insulin signaling, adipocytokine signaling, PPAR signaling, and oxidative phosphorylation.

The FoxO transcription factors constitute a vital protein family regulating various biological processes, such as cell cycle progression, apoptosis [83,84]. Activation of the FoxO signaling pathway plays a pivotal role in maintaining metabolic homeostasis [85]. Exposure to high environmental ammonia in grass carp significantly alters the FoxO signaling pathway, which induces oxidative stress [86]. Similarly, under hypoxic conditions, crucian carp (*Carassius auratus*) exhibit significant enrichment of upregulated genes in the FoxO signaling pathway [87]. In turbot, thermal stress enhances key hub genes in the FoxO signaling pathway [88]. The findings from the current project suggested that H_2_O_2_ activated FoxO signaling via upregulation of IRS, AMPK, and SGK, subsequently increasing expression of BNIP3, Gadd45, and Atrogin-1, ultimately triggering muscle autophagy, oxidative stress, and DNA damage. Our findings corroborated this stress-response paradigm, demonstrating that H_2_O_2_-induced oxidative stress upregulates foxo3 expression along with its downstream targets (BNIP3, Gadd45, and Atrogin-1) in carp muscle. Notably, oxidative stress can upregulate BNIP3 expression, thereby activating autophagy and exerting cytoprotective effects [89]. Additionally, Gadd45 is primarily involved in biological processes such as cell cycle regulation, oxidative stress response, and apoptosis [90,91]. Therefore, we reasonably hypothesize that the upregulation of these related genes triggers protective mechanisms to counteract oxidative stress.

Lipid, glucose, and protein metabolism are principally modulated through the insulin signaling pathway [92]. The insulin signaling pathway exhibits biphasic responses to oxidative stress. Acute moderate stress typically induces transient activation as an adaptive response, whereas chronic or severe stress causes pathway suppression and downstream signaling impairment [93]. In liver common carp, H_2_O_2_ exposure induces dysregulation of insulin signaling that may subsequently disrupt protein synthesis and metabolic homeostasis [94]. Our data demonstrated that H_2_O_2_ exposure altered the insulin signaling pathway in common carp muscle, as evidenced by the upregulation of 14 key genes (including *irs1*, *irs2*, and *calml4a*), suggesting potential impacts on lipid biosynthesis and glucose metabolic processes. This likely represents an adaptive response to oxidative stress, where increased lipidogenesis and glucose metabolism provide essential fatty acids and energy to counteract H_2_O_2_-induced cellular damage.

The adipocytokine signaling pathway comprises a cascade of biochemical reactions mediated by hormones and cytokines synthesized and secreted by adipocytes. These molecules regulate physiological processes through binding to cell surface receptors, playing pivotal roles in energy homeostasis, inflammatory responses, and immune regulation [38,95]. Accumulating evidence demonstrates that the adipocytokine pathway serves as a crucial molecular mechanism for aquatic species to counteract oxidative stress, as observed in cold-stressed spotted sea bass (*Lateolabrax maculatus*) [96], density-stressed common carp [95], and copper-exposed rainbow trout [97]. Bisphenol A exposure has been shown to upregulate adipocytokine pathway in zebrafish (*Danio rerio*), disrupting lipid homeostasis through enhanced lipid biosynthesis and transport coupled with suppressed catabolic processes [98]. The alteration of the adipocytokine signaling pathway was observed following H_2_O_2_ treatment, with the current study revealing that 11 associated genes were markedly upregulated. The results suggested that stress-induced enhancement of lipid metabolism in carp muscle represents an adaptive response to mitigate H_2_O_2_-mediated oxidative damage.

The PPAR signaling pathway represents a crucial transcriptional regulatory network, with PPARα playing pivotal roles in lipogenesis and lipid metabolism [99]. In juvenile turbot, thermal stress activated PPAR signaling through fatty acid interactions, demonstrating the protective role of lipid metabolism in stress adaptation [100]. Zebrafish exposed to permethrin (Per) exhibited oxidative stress-mediated activation of the KRAS-PPAR-GLUT axis, resulting in lipid metabolic disruption [101]. Various stressors, including microplastics [102], acute nitrite [103], and copper exposure [104], have been shown to dysregulate PPAR signaling in aquatic species, leading to metabolic imbalances. Our study revealed an upward trend in the PPAR pathway in common carp muscle following H_2_O_2_ exposure, which may be an adaptive mechanism to counteract H_2_O_2_-induced oxidative stress.

Oxidative phosphorylation, the primary ATP-generating process in cells, is driven by proton and electron gradients across the mitochondrial inner membrane [105]. Oxidative phosphorylation serves as the central hub for energy metabolism in the stress responses of aquatic organisms. Stress-induced metabolic suppression in these species is generally linked to substantial inhibition of ATP production pathways [106,107,108]. Under high ammonia stress, *E. sinensis* enhances oxidative phosphorylation to meet energy demands [109]. In contrast, copper exposure disrupts normal electron transport and mitochondrial oxidative phosphorylation in oriental river prawn larvae, thereby suppressing energy production [110]. Additionally, nanoplastic exposure induces dysregulation of oxidative phosphorylation in the red swamp crayfish, leading to abnormal energy metabolism [111]. Our study demonstrated an upward trend in oxidative phosphorylation in muscle following H_2_O_2_ exposure, in agreement with the findings reported by Jia et al. [112]. Oxidative stress induced by H_2_O_2_ may activate energy metabolism reprogramming in common carp muscle tissue, thereby enhancing its adaptive response to environmental stressors.

Metabolomic analysis revealed that H_2_O_2_ exposure altered pathways including the biosynthesis of alkaloids derived from histidine and purine, glyoxylate and dicarboxylate metabolism, and carbon metabolism. It also induced changes in the abundance of metabolites such as lipids, amino acids, and nucleotides, thereby affecting energy metabolism and lipid metabolism processes in muscle. Although integrated analysis did not identify any common significantly enriched pathways shared between the metabolome and transcriptome, the significantly altered pathways in the transcriptome were closely associated with energy metabolism and lipid metabolism. These included oxidative phosphorylation, adipocytokine signaling pathway, insulin signaling pathway, and PPAR signaling pathway. These results demonstrate that H_2_O_2_ exposure induces metabolic reprogramming at the molecular level by reshaping metabolic pathways in muscle, leading to alterations in metabolites, including amino acids, lipids, and nucleotides. The specific regulatory networks involved require further investigation.

Although the composition of macronutrients (e.g., amino acids and fatty acids) remained statistically unchanged, significant perturbations were detected at both the transcriptomic and metabolomic levels, including marked upregulation of certain metabolites and alterations in metabolic functions and pathways. This dissociation between apparent compositional stability and profound molecular dynamics highlights the complex regulatory mechanisms underlying the organism’s response to oxidative stress. The initial impact of oxidative stress on muscle quality manifests more prominently as dysfunction in molecular activities and disruption of metabolic pathways, rather than as immediate large-scale changes in macronutrient content [113,114].

The findings of this study provide a scientific basis for the safe and standardized use of H_2_O_2_ in aquaculture. Furthermore, as a typical exogenous stressor, H_2_O_2_ can be effectively employed to assess the physiological and metabolic responses of farmed fish under stress conditions, thereby offering a novel approach for health monitoring and stress resistance evaluation. Based on these findings, further efforts can be directed toward developing targeted nutritional regulation products and management strategies. It furthermore establishes a theoretical foundation for developing antioxidant intervention approaches. For instance, dietary supplementation with specific additives such as proanthocyanidins [57], which may protect mitochondrial function and alleviate lipid peroxidation damage, could offer targeted mitigation of metabolic disturbances induced by oxidative stress.

Limitations of the study: (1) This study employed only a single concentration of H_2_O_2_ treatment, which presents a limitation in elucidating the concentration–effect relationship. (2) Although no mortality or overt behavioral abnormalities were observed in common carp during the trial, visual assessment may introduce subjective bias. In subsequent studies, automated behavioral analysis systems will be utilized to enable objective and quantitative evaluation, allowing for more precise detection of stress-associated behavioral changes. (3) The integrated metabolomic and transcriptomic analysis did not reveal any common significantly enriched pathways. This result may be attributed to the single treatment concentration and exposure duration used in the current experimental design. The underlying mechanisms require further clarification through experiments incorporating multiple concentration–time gradients.

## 5. Conclusions

This study provides evidence that common carp adapt to H_2_O_2_-induced oxidative stress in muscle through altered metabolic pathways. While H_2_O_2_ exposure showed no significant effects on the content and composition of amino acids and fatty acids in carp muscle, metabolomic analysis revealed substantial modifications in lipid, amino acids, and nucleotides. These changes reflect metabolic reprogramming triggered by oxidative stress through the modulation of energy metabolism, lipid metabolism and antioxidant defense systems. Transcriptomic profiling further demonstrated significant alterations in key signaling pathways, including insulin signaling, adipocytokine signaling, oxidative phosphorylation, PPAR signaling, and FoxO signaling. These findings may suggest that muscle tissue may adapt to oxidative stress by regulating ATP production and lipid biosynthesis. As the first study to integrate metabolomic and transcriptomic analyses in examining the muscular adaptive response of common carp to H_2_O_2_-induced stress, this work provides novel insights into the toxicological mechanisms of H_2_O_2_ in aquaculture. The findings establish a theoretical basis for the development of antioxidant interventions and offer important implications for improving health management and targeted nutritional strategies in farmed fish.

## Figures and Tables

**Table 1:** Hydrolyzed amino acid composition in *C. carpio* muscle (g/100 g, wet weight).

Amino Acids (g/100 g, ww)	Groups		Percent Difference	*p*-Value
	NC	HT		
Thr	0.62 ± 0.02	0.60 ± 0.02	−3.23%	0.364
Val	0.72 ± 0.03	0.68 ± 0.02	−5.56%	0.233
Met	0.38 ± 0.02	0.35± 0.01	−7.89%	0.294
Ile	0.65 ± 0.02	0.61 ± 0.02	−6.15%	0.232
Leu	1.17 ± 0.04	1.11 ± 0.03	−5.13%	0.276
Phe	0.64 ± 0.03	0.60 ± 0.02	−6.25%	0.294
Lys	1.43 ± 0.05	1.37 ± 0.04	−4.20%	0.375
His	0.57 ± 0.03	0.53 ± 0.02	−7.02%	0.348
Arg	0.84 ± 0.03	0.81 ± 0.02	−3.57%	0.386
Asp	1.40 ± 0.06	1.31 ± 0.05	−6.43%	0.233
Ser	0.47 ± 0.02	0.44 ± 0.01	−6.38%	0.199
Glu	1.85 ± 0.07	1.67 ± 0.05	−9.73%	0.082
Gly	0.62 ± 0.02	0.60 ± 0.01	−3.23%	0.583
Ala	0.88 ± 0.03	0.85 ± 0.03	−3.41%	0.484
Cys	0.16 ± 0.02	0.13 ± 0.01	−18.75%	0.388
Tyr	0.46 ± 0.02	0.44 ± 0.01	−4.35%	0.398
Pro	0.47 ± 0.02	0.45 ± 0.01	−4.26%	0.434
EAAs	5.61 ± 0.21	5.31 ± 0.16	−5.35%	0.295
CEAAs	1.40 ± 0.06	1.34 ± 0.03	−4.29%	0.352
NEAAs	6.31 ± 0.25	5.90 ± 0.17	−6.50%	0.218

EAAs, essential amino acids; CEAAs, conditionally essential amino acids; NEAAs, non-essential amino acids; ww, wet weight. All data were calculated as mean ± SEM (*n* = 5).

**Table 2:** Hydrolyzed fatty acid composition in *C. carpio* muscle (mg/100 g, wet weight).

Fatty Acids (mg/100 g, ww)	Groups		Percent Difference	*p*-Value
	NC	HT		
C16:0	103.56 ± 4.23	99.84 ± 3.31	−3.59%	0.508
C16:1	4.98 ± 0.87	4.12 ± 1.09	−17.27%	0.555
C18:0	39.50 ± 1.12	38.86 ± 0.68	−1.62%	0.639
C18:1n9c	133.08 ± 12.85	121.56 ± 9.25	−8.66%	0.488
C18:2n6c	138.88 ± 10.16	125.52 ± 7.99	−9.62%	0.441
C18:3n3	7.62 ± 0.79	6.82 ± 0.59	−10.50%	0.832
C20:1	6.44 ± 0.65	6.24 ± 0.29	−3.11%	0.785
C20:2	5.92 ± 0.37	6.02 ± 0.27	1.69%	0.884
C20:3n6	14.20 ± 0.69	14.08 ± 0.40	−0.85%	0.459
C22:1n9	3.12 ± 0.82	3.34 ± 1.94	7.05%	0.331
C20:4n6	35.80 ± 3.31	32.84 ± 1.87	−8.27%	0.872
C22:6n3	17.70 ± 1.48	18.08 ± 1.75	2.15%	0.484
Total SFA	148.06 ± 4.89	138.70 ± 3.37	−6.32%	0.481
Total MUFA	147.62 ± 13.46	135.26 ± 9.94	−8.37%	0.188
Total PUFA	220.12 ± 8.36	203.36 ± 8.09	−7.61%	0.858
Total UFA	367.74 ± 45.62	338.62 ± 37.77	−7.91%	0.304

SFA, saturated fatty acid; MUFA, monounsaturated fatty acid; PUFA, polyunsaturated fatty acid; ww, wet weight. All data were calculated as mean ± SEM (*n* = 5).

**Table 3:** Quality assessment of RNA-seq data.

Samples	Raw Data (bp)	Clean Data (bp)	Total Reads	Q20 (%)	Q30 (%)	GC (%)	Total Mapped
NC1	6,386,695,500	6,285,991,447	41,054,402	98.12	95.81	50.02	93.73%
NC2	6,411,411,600	6,260,831,140	41,489,560	97.72	95.09	49.30	92.93%
NC3	5,782,143,300	5,679,862,256	45,412,678	97.89	95.41	49.94	93.79%
NC4	7,055,086,200	6,901,618,956	48,139,874	97.80	95.26	49.77	93.40%
NC5	7,412,127,000	7,249,214,727	37,557,260	97.90	95.43	50.04	93.40%
HT1	7,291,706,100	7,100,651,939	46,258,780	97.64	94.88	49.87	93.08%
HT2	7,417,197,738	7,198,571,166	47,574,450	97.29	94.22	49.43	92.21%
HT3	5,718,504,300	5,580,781,347	36,795,230	97.66	94.90	49.45	92.79%
HT4	6,525,954,900	6,357,279,854	42,060,676	97.60	94.90	49.05	92.27%
HT5	7,097,538,300	6,948,760,394	45,946,326	97.81	95.23	49.49	92.79%

All clean reads satisfied Q20/Q30 criteria (Q scores ≥ 20/30), GC, GC content in clean reads.

## Data Availability

All data are contained within the main manuscript. The raw transcriptomic sequencing data have been deposited in the NCBI database (PRJNA1300964).

## Supplementary Materials

The following supporting information can be downloaded at: https://www.mdpi.com/article/10.3390/antiox14091115/s1, Table S1: Specific primer sequences for qPCR in the study.
