# Supplementary material for: Integrated Metabolomics and Transcriptomics Reveals Metabolic Pathway Changes in Common Carp Muscle Under Oxidative Stress
Source: Antioxidants (Basel). 2025 Sep 14;14(9):1115. doi: 10.3390/antiox14091115 (PMC12466421; doi:10.3390/antiox14091115)
Supplement: Supplementary file 1 [file antioxidants-14-01115-s001.zip › antioxidants-3848893-supplementary.pdf]

Table S1. Specific primer sequences for qPCR in the study.

Gene	Primer sequence (5′-3′)
β-actin	F: ATCCGTAAAGACCTGTATGCCA<br>R: GGGGAGCAATGATCTTGATCTTCA
18S rRNA	F: GAGTATGGTTGCAAAGCTGAAAC<br>R: AATCTGTCAATCCTTTCCGTGTCC
succinate dehydrogenase (ubiquinone) flavoprotein subunit (*sdha*)	F: AGTTGGACTTCCCGTGTGTTT<br>R: CACCGCTGGCAGAGCTTTAG
cytochrome c oxidase subunit 8 (*cox8*)	F: CAAGATCGGACCTGGGCAAAGT<br>R: TTGACCGTGACGGAGGTGTT
NADH dehydrogenase (ubiquinone) 1 alpha subcomplex subunit 12(*ndufa12*)	F: GTGTTTGGAGTCCGTCAGCTA<br>R: CAATACCCACCAAGGCTCCA
cytochrome c oxidase subunit 6a (*cox6a*)	F: CCAAAACACCTGACCACCC<br>R: CAGGTCCTTGCTCCTCCTTC
NADH dehydrogenase (ubiquinone) 1 beta subcomplex subunit 7 (*ndufb7*)	F: AGACCGGAAACATCAGAGGG<br>R: TGATGTAACTCCGCACGAGG
ATPase subunit g (*atp20*)	F: TCTGAGGAACGGACTTGTTG<br>R: GCAAAGAGAACAAGCAAACAGG
insulin receptor substrate 2 (*irs2*)	F: TTGCCAGCTGTGATTCCCTT<br>R: AGACTGGCTCTGTTTAGTCTTT
growth arrest and DNA damage inducible 45b (*gadd45b*)	F: CAGCGGCATATTCGTGGC<br>R: ACCGAGTCCATTCTTTCCGT
F-box protein 32 (*fbxo32*)	F: CACATCCTGTTTTGGAAGGATACAA<br>R: AATGCAGCGCAACCTAGAACT
forkhead box protein O1 (foxo1-a)	F: CGAGCAGCAAAGAAAAAGGGTG<br>R: GTCCAGGCGTCAAAGTCATC
GABA(A) receptor-associated protein (atg8)	F: GCACCCCATCCAAGATGTCTA<br>R: GGATGTTTGGTGCGGACTCT
acetyl-CoA carboxylase / biotin carboxylase 2 (acacb)	F: CAACACATCGGTCAGTAGCCT<br>R: TGCTCCGCTGAGAAATGTCC
protein phosphatase 1 regulatory subunit 3Ab (ppp1r3ab)	F: ATCTTCACCTGTGCCGAGAC<br>R: CGAAGGCATCAGCAAAGGAC
ras homolog gene family member Q (tc10)	F: GGGCGAATGACAAGATGAACT<br>R: TCATTAGAAAGGGTGTCTGTTCTC
suppressor of cytokine signaling 3 (socs3)	F: GGGGTGGCTACTTACCGTTC<br>R: GTAACAGGCCTCTCGTCTGG
protein phosphatase 1 regulatory subunit 3 (ppp1r3)	F: TAAACCGCTGCCATTGTGTG<br>R: TCTTCAACCGGAAATCGGCT
aquaporin 7(aqp7)	F: CCGCCCAGTGACAACCATAC<br>R: GGTGCCATATGGTCTTGTATCCT
apolipoprotein A-I (apoa1b)	F: TTTCGCAGCCGTCCAAAAGG<br>R: TGGATGACGATGTTTCAGGTGT
long-chain acyl-CoA synthetase(*acsl*)	F: ACAAGACACCGTTCTCCGTAA<br>R: GGTTGGAGTGTATCTGTCAGGC
